# Lentivirus-mediated RNA interference targeting HMGB1 modulates AQP1 to reduce pain induced by chronic compression of the dorsal root ganglia

**DOI:** 10.3389/fphar.2024.1469223

**Published:** 2024-09-18

**Authors:** Jinlu Li, Kaihong Yang, Fuchao Yao, Hui Wei

**Affiliations:** ^1^ School of Nursing and Rehabilitation, Cheeloo College of Medicine, Shandong University, Jinan, China; ^2^ Rehabilitation Center, Qilu Hospital of Shandong University, Jinan, China

**Keywords:** HMGB1, CCD, AQP1, neuropathic pain, gene therapy, RNA interference

## Abstract

**Backgrounds:**

Neuropathic pain (NP) is a kind of chronic pain that has attracted much attention in clinical practice, characterized by high morbidity, complex mechanisms, and difficulties in clinical treatment, with which the activation of High mobility group box 1 (HMGB1) is closely related. The aim of this study was to investigate the effects of lentivirus-mediated RNA interference gene therapy targeting HMGB1 on neuropathic pain in rats with chronic dorsal root ganglion compression (CCD) and its specific mechanisms, so as to explore new pharmacological targets.

**Methods:**

Adult male Wistar rats were surgically subjected to chronic compression of the dorsal root ganglia (CCD). Behavioral tests were performed by calculating the paw withdrawal mechanical threshold (PWMT) and the thermal paw withdrawal latency (TPWL). Co-immunoprecipitation (CO-IP) was used to clarify protein interactions. Gene silencing was induced by injecting lentivirus expressing HMGB1 short hairpin RNA (shRNA) into rats. An LPS-inflammation-stimulated rat astrocyte model was established to validate the animal experiment results further. Western blot analysis and real-time quantitative PCR were used to detect pathway protein expression.

**Results:**

After first establishing the rat CCD model, both PWMT and PTWL were significantly reduced in rats, indicating that the model construction was successful. After lentiviral silencing of HMGB1 expression, NP was significantly alleviated in CCD rats. CO-IP experiments showed a link between HMGB1 and AQP1; After silencing HMGB1 expression, the expression of AQP1 was significantly reduced, and HMGB1 was able to modulate the effect of AQP1 on NP. Further use of an inhibitor of the HMGB1 receptor showed that after inhibition of RAGE, AQP1 was significantly reduced; HMGB1 may regulate AQP1 through its receptor RAGE to affect NP. Silencing of HMGB1 resulted in a significant decrease in NF-κB, and HMGB1 affects the inflammatory pathways it mediates. After silencing AQP1, NF-κB also decreased significantly, indicating that AQP1 is an upstream regulator of NF-κB.

**Conclusion:**

Lentivirus-mediated RNA interference (RNAi) silencing targeting HMGB1 may play a key role in the development of neuropathic pain in rats by regulating AQP1 expression via RAGE and ultimately activating NF-κB.

## 1 Introduction

Neuropathic pain (NP) is a chronic pain condition resulting from lesions of the somatosensory nervous system. NP is associated with high rates of morbidity and severely reduces the quality of life in these individuals as the financial burden on the patient and the risk of depression are compounded by the negative physiological and psychological effects experienced by their pain (Cherif et al., 2020). Its pathogenesis is complex, with peripheral sensitization, central sensitization, activation of spinal cord glial cells, and the development of inflammatory responses all contributing to the development of NP (Thouaye and Yalcin, 2023). There are three main manifestations of neuropathic pain, allodynia and hyperalgesia/spontaneous pain (Bannister et al., 2020). The identification of novel pharmacological targets which may provide new insights and assist in the clinical treatment and management of NP is sorely warranted.

HMGB1 represents an essential molecule in the development of NP (Wu and Li, 2023). This non-histone protein is actively secreted by reactive astrocytes and microglia in the central nervous system (CNS) or passively released from necrotic cells after injury. As a result, HMGB1 functions as a late-phase inflammatory factor (Chen et al., 2022). The pro-inflammatory effects of HMGB1 are primarily mediated by the activation of receptors, including the receptor for advanced glycosylation end-products (RAGE) and TOLL-like receptors (e.g., TLR4), which are expressed on the surfaces of cell membranes within a multitude of cell types. Finally, these receptor-mediated pro-inflammatory effects of HMGB1 activate the nuclear transcription factor, NF-κB, which is ultimately responsible for the observed inflammatory effects (Yang et al., 2020). HMGB1 is believed to play a pivotal role in nociceptive hypersensitivity through activation of its receptors RAGE and TLR4, as demonstrated in chemotherapy or chronic constrictive injury (CCI) animal models of NP (Morioka et al., 2019; Zhao et al., 2020; Moraes et al., 2024). Therefore, targeting HMGB1 can be considered an important target site for use in NP therapy. However, the mechanisms of HMGB1 action in NP development are complex and diverse and its downstream targets remain to be clarified. Gene therapy offers a therapeutic approach with great potential for treating diseases that drugs can control but not correct (Sudhakar and Richardson, 2019). For example, the lentivirus-mediated expression of short hairpin RNA (shRNA) represents a form of gene therapy designed to locally knockdown the expression of specific genes. This procedure can serve as a means to evaluate the mechanisms of gene action and achieve long-term, stable therapeutic efficacy (Ichim et al., 2004; Germain et al., 2023).

Aquaporin 1 (AQP1), a water transport protein mediating passive transmembrane fluid transport, plays an important role in maintaining a homeostatic balance between intra- and extra-cellular fluid levels. Results from recent studies have revealed that AQP1 may serve as a potential marker of inflammation in the pathophysiologic development of inflammatory diseases (Gao et al., 2024; Kalita and Das, 2024). AQP1 may also function as a target in the treatment of NP through its capacity to regulate TRPV4, as demonstrated in a rat model of chronic compression of the dorsal root ganglia (CCD) (Wei et al., 2020). We have hypothesized that AQP1 plays an equally important role in inflammatory signaling and is involved in the pathophysiological processes of NP.

As HMGB1 may represent a critical pharmacological target for use in the treatment of NP, it is important to establish the mechanisms of HMGB1. Although there have been some current investigations directed at examining the role of HMGB1 in NP, its downstream targets remain unclear. Moreover, the link between HMGB1 and AQP1 currently lacks evidence and whether any relationship exists between the two as related to NP has yet to be established. Based on the information presented above, an investigation into the use of lentivirus-mediated gene therapy to explore some of the specific mechanisms of HMGB1 in NP is clearly warranted. Our study has the potential to provide a new pharmacological target and theoretical basis for the treatment of NP, as well as identify new perspectives and directions for the use of HMGB1 in the treatment of NP.

## 2 Materials and methods

### 2.1 Animals

This study was approved for conducting the animal experiments performed by the ethics committee of the School of Nursing and Rehabilitation of Shandong University. It was designed according to the principle of “Replacement, Reduction and Refinement (3Rs)” and complied with the guidelines for ethical standards of the International Association for the Study of Pain, with all efforts being made to minimize the pain experienced by the animals and to reduce the number of animals used in the experiments.

Adult male Wistar rats (180–200 g body weight) were provided by the Laboratory Animal Center of Shandong University. All rats were housed under standard laboratory conditions (20°C ± 2°C, 12 h light/dark cycle) at the Animal Model Experimental Center of Shandong University. They were provided with free access to standard laboratory chow and tap water and were allowed to acclimate to this diet for 1 week prior to use in the experiments.

### 2.2 Chronic dorsal root ganglion compression (CCD) animal model

Following anesthetization with sodium pentobarbital (Nembutal, 50 mg/kg i.p.), the skin, subcutaneous tissue, muscle, and fascia were dissected in layers to expose the L3-4 and L4-5 intervertebral foramina bilaterally. A U-shaped steel rod was inserted to exert constant pressure on the dorsal root ganglia (DRG) and adjacent nerve roots on the right (injury) side while the left side remained uninjured (Wei et al., 2020). Rats in the sham surgery group underwent an identical surgery, however, the steel rod was not inserted. Rats demonstrating autophagy, a sensory deficiency or any generalized handicaps were eliminated from the study.

### 2.3 Chemicals

TAK-242 (TLR4 inhibitor) and FPS-ZM1 (RAGE inhibitor) were purchased from the GlpBio Company (Montclair, CA). Use of drugs in the animal experiments were dissolved in 10% DMSO + 90% corn oil while those used in the cellular experiments were dissolved in DMSO and then diluted to the target concentration using the complete culture medium for dosing. The following primary antibodies were used: rabbit anti-HMGB1 antibody (20 μg, CUSABIO), rabbit anti-AQP1 antibody (20 μL, BOSTER BIOLOGICAL TECHNOLOGY), rabbit anti-RAGE antibody (10 μL, MedChemExpress), rabbit anti-GAPDH antibody (100 μL, BOSTER) and mouse anti-TLR4 antibody (20 μL, proteintech).

### 2.4 Generation of lentivirus expressing HMGB1 and AQP1 shRNAs

HMGB1-specific target sequences were chosen based on online shRNA tools provided by Invitrogen (www.invitrogen.com/rnai) using an HMGB1 reference sequence. The following target sequences were used for HMGB1:5′- TCT​GTA​ATT​TGA​GGA​GGA​ATA -3′ (HMGB1-shRNA-#1), 5′- CCC​TAC​TAA​AGA​CCT​GAG​AAT -3′ (HMGB1-shRNA-#2), and 5′- AAA​CTA​ATA​ATT​GCA​GAG​GTT -3′ (HMGB1-shRNA-#3), followed by the chemically synthesized shRNA (GeneChem, Shanghai, China) with the lentiviral vector then constructed per the Invitrogen lentiviral vector protocol. A scrambled sequence (5′- TTC​TCC​GAA​CGT​GTC​ACG​T-3′) was used as the negative control shRNA. The following target sequences were used for AQP1:5′-CAGGGTGGAGATGAAGCCCAA -3′ (AQP1-shRNA-#1) with the scrambled sequence (5′- TTC​TCC​GAA​CGT​GTC​ACG​T-3′) used as the negative control shRNA. Vectors expressing specific shRNAs were confirmed via sequencing.

### 2.5 Astrocyte cultures

Rat astrocyte cell lines (CTX) were purchased and cultured in a complete medium (1% double antibody + 10% fetal bovine serum + 89% basal medium). The LPS powder (10mg, Meilun Bio) was dissolved in PBS with the LPS and then administered at an appropriate cell density of 1 μg/mL. After LPS stimulation, TAK-242 (5 mg/kg, 24 h) or FPS-ZM1 (1 mg/kg, 48 h) was administered with the optimal concentrations and times used based on results from previous and pilot studies.

### 2.6 Behavioral testing

Mechanical pain sensitivity was assessed using the mechanical withdrawal threshold (MWT) index in response to the Von Frey Fibers mechanical withdrawal reflex as achieved using the Von Frey Fibers mechanical nociceptive stimulator (BME-404, Biomedical Engineering Institute of Chinese Academy of Medical Sciences). The effect of thermal nociceptive sensitization in rats was determined by the paw withdrawal latency (PWL) response to thermal radiation stimulation as measured using a thermal nociceptive stimulator (BME-410C, Biomedical Engineering Institute of Chinese Academy of Medical Sciences). The gait of these rats was evaluated and those showing autophagy, sensory deficits or disabilities were excluded from the experiments.

Behavioral tests were performed on the ipsilateral hind paw before surgery and on postoperative days 0, 3, 4, and 7. The expression of HMGB1 was inhibited at 24 h after lentivirus injection and to maintain this suppression of HMGB1, the HMGB1 lentivirus was continuously injected for 3 days. These tests were repeated five times with a minimum of a 5-min interval between tests with the average value calculated and then used for statistical analyses.

### 2.7 Western blotting

The L4-L5 spinal cord on the injured side within each group was dissected and the cells were harvested. The extracted proteins were separated by 10% SDS-PAGE according to molecular weight, followed by membrane transfer; the transferred membranes were incubated with 5% skimmed milk for 1–2 h at room temperature. After adding the appropriate primary antibody and incubating overnight at 4°C, the polyfluoroethylene membrane was incubated with anti-rabbit or anti-mouse peroxidase (HRP)-linked secondary antibody (1: 5000, Zhongshan Goldenbridge, Beijing, China) for 1 h. Binding was determined using the chemiluminescence HRP method (Millipore, Billerica, MA, United States). Protein expressions of HMGB1, AQP1, RAGE, TLR4, NF-κB, and GAPDH were analyzed using ImageJ software.

### 2.8 Real-time quantitative PCR

Total RNA from the L4-L5 spinal cord samples of each group of rats was extracted using the Trizol method. Cellular RNA was extracted using the RNA Extraction Kit (Feijie Biotechnology, Shanghai) and was reverse transcribed to form the stable cDNA. The following primers were used to amplify the fragments of each factor (Table 1). Instrument control, automated data collection and data analyses were performed using the LightCycler software program, version 4.0. The 2^−ΔΔCT^ method was used to analyze the data.

**TABLE 1:** Contains a summary of the Primer Sequence List.

Name	Primer sequences
Rattus-AQP1-78 F	ATT​CAT​TGG​GAG​TGC​CCT​GG
Rattus-AQP1-78 R	ATG​CGG​TCT​GTA​AAG​TCG​CT
Rattus-HMGB1-98F	CCT​AAG​AAG​CCG​AGA​GGC​AA
Rattus-HMGB1-98R	AAG​TTG​ACA​GAA​GCA​TCC​GGG
Rattus-TLR4-197F	CCA​GAG​CCG​TTG​GTG​TAT​CT
Rattus-TLR4-197R	AGA​AGA​TGT​GCC​TCC​CCA​GA
Rattus-NF-κB-123F	TCC​TGC​TTA​CGG​TGG​GAT​TG
Rattus- NF-κB −123R	CTG​TCG​TCA​CTC​TTG​GCA​CA
Rat-RAGE-151F	GGT​CAC​AGA​AAC​CGG​TGA​TGA​A
Rat-RAGE-151R	TCT​GGG​TTG​TCG​TTT​TCG​CC

### 2.9 Co-immunoprecipitation (CO-IP)

A sample (75 µL) of protein A/G immunoprecipitation (IP) beads was obtained and washed with 0.5% Triton X-100 in phosphate-buffered saline (PBS). Flag antibody was then added and incubated at 4°C overnight. The protein A/G IP beads and Flag antibody complex was resuspended in 150 µL of co-immunoprecipitation (CO-IP) binding buffer, separated by a magnetic rack, washed with CO-IP wash buffer, and the supernatant was discarded. The beads were then resuspended in 150 µL of CO-IP wash buffer. RIPA lysate (500 µL), containing a protease inhibitor cocktail, was added to each group of cells. The cells were lysed on ice for 30 min, centrifuged at 4°C for 10 min at 12,000 rpm and the resultant supernatant was collected. Next, 50 µL of the Protein A/G IP Bead + Flag Antibody Complex was added to each cell lysate and incubated at 4°C overnight. The beads were then washed again and the supernatant discarded. Finally, 100 µL of 1X SDS-PAGE loading buffer was added to the precipitate and boiled at 100°C for 10 min, followed by Western blot (WB) assay.

### 2.10 Statistical analysis

All quantitative results are expressed as mean ± SEM. Two-way analysis of variance (ANOVA) was used to compare the behavioral data of the different groups. One-way repeated measures analysis of variance (ANOVA) was used to analyze the differences in the expression levels of the factors. Multiple comparisons were used to identify significant differences between specific groups. All statistical analyses were performed using Prism 9 (GraphPad Software, San Diego, CA) and *p* < 0.05 was considered statistically significant.

## 3 Results

### 3.1 Verification of HMGB1 mRNA interference treatment

To assess the knockdown efficiency of lentivirus in the *in vitro* studies, rat astrocytes were infected with Lv-shRNA-HMGB1-#1, Lv-shRNA-HMGB1-#2, Lv-shRNA-HMGB1-#3 and a negative control shRNA (Lv-shRNA-NC).

**FIGURE 1:**
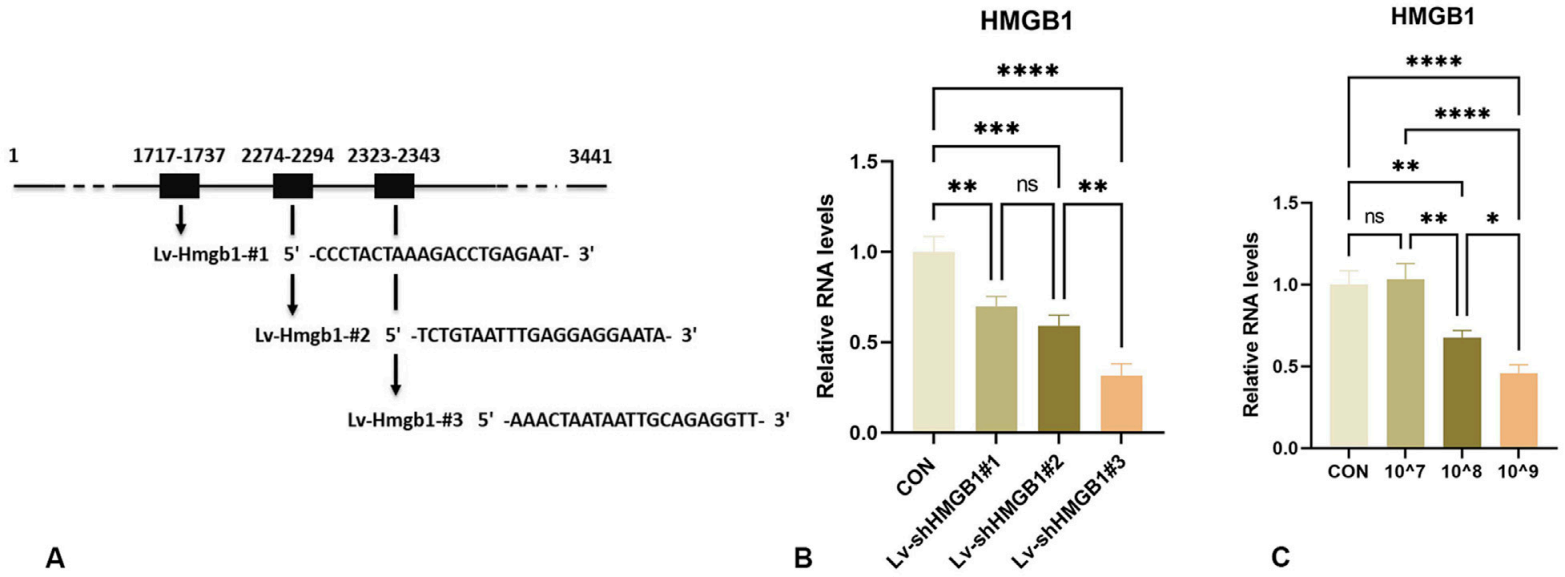
Design and characterization of HMGB1-interfering lentiviruses **(A)** Schematic design of shRNA targeting HMGB1 mRNA. **(B)** Real-time PCR analysis of HMGB1 RNA levels in rat astrocytes infected with lentivirus expressing HMGB1 shRNA. **(C)** qRT-PCR analysis of HMGB1 gene expression in the spinal cord of rats injected with different titers. ****p < 0.0001 ***p < 0.001 **p < 0.01*p < 0.05.

As compared with that of the CON group, Lv-shRNA-HMGB1-#1, Lv-shRNA-HMGB1-#2 and Lv-shRNA-HMGB1-#3 were all able to effectively reduce HMGB1 gene expression levels (Figures 1A, B). Maximal levels of HMGB1 downregulation were achieved with Lv-shRNA-HMGB1-#3, as compared with that of the other two lentiviruses (*p* < 0.01). Therefore, Lv-shRNA-HMGB1-#3 was selected for use in subsequent studies.

### 3.2 Optimizing Lv-shRNA-HMGB1 concentration for use *in vivo*

To minimize the number of trials and the number of animals used, knockdown efficiencies of the lentivirus were tested using different titers (10^7, 10^8 and 10^9 TU/mL). As shown in Figure 1C, a concentration of 10^9 TU/mL significantly downregulated HMGB1 gene expression in the rat spinal cord (Figure 1C). Therefore, this concentration was selected for subsequent experiments.

### 3.3 Temporal changes in mechanical and thermal pain thresholds in CCD rats

**FIGURE 2:**
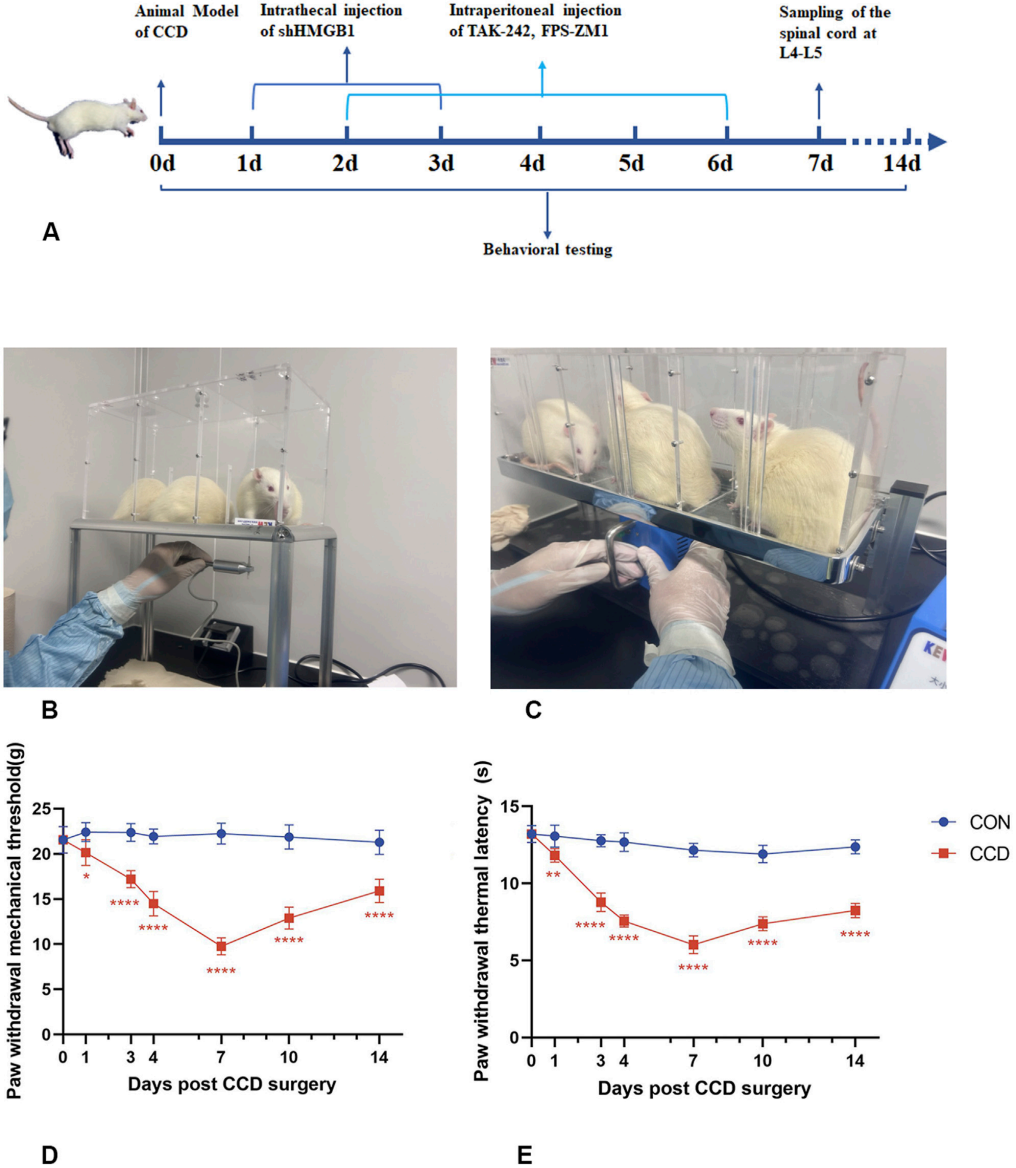
Temporal changes in mechanical and thermal pain threshold responses of CCD rats **(A)** Experimental schedule chart. **(B)** Actual test chart for mechanical pain threshold in rats. **(C)** Actual test chart for thermal pain threshold in rats. **(D)** Line graph of mechanical pain threshold in rats. **(E)** Line graph of thermal pain threshold in rats. N = 5 per group ****p < 0.0001 ***p < 0.001 **p < 0.01*p < 0.05 vs. CON.

To investigate the temporal changes in mechanical and thermal pain thresholds, rats were repeatedly tested over 14 days (Figures 2A–C). In the CON group, no statistically significant differences were obtained for either mechanical or thermal pain thresholds in the ipsilateral hind paw over this 14-day period (*p* > 0.05). All CCD rats showed normal motor behavior, suggesting that the surgery did not impair their basic motor functions. On days 3 and 4 post-CCD surgery, mechanical and thermal pain thresholds in CCD rats were significantly decreased as compared with those in the CON group. Mechanical and thermal pain thresholds in CCD rats achieved their lowest levels on day 7 post-CCD with these levels being significantly lower than that of the CON group (*p* < 0.0001). On day 14 after CCD surgery, mechanical and thermal pain thresholds of CCD rats showed a gradual increase (Figures 2D, E). These results demonstrate that the peak period of nociceptive sensitization in CCD rats was on day 7 post-surgery and therefore this period was selected for subsequent studies.

### 3.4 HMGB1 knockdown reduces NP in CCD rats

**FIGURE 3:**
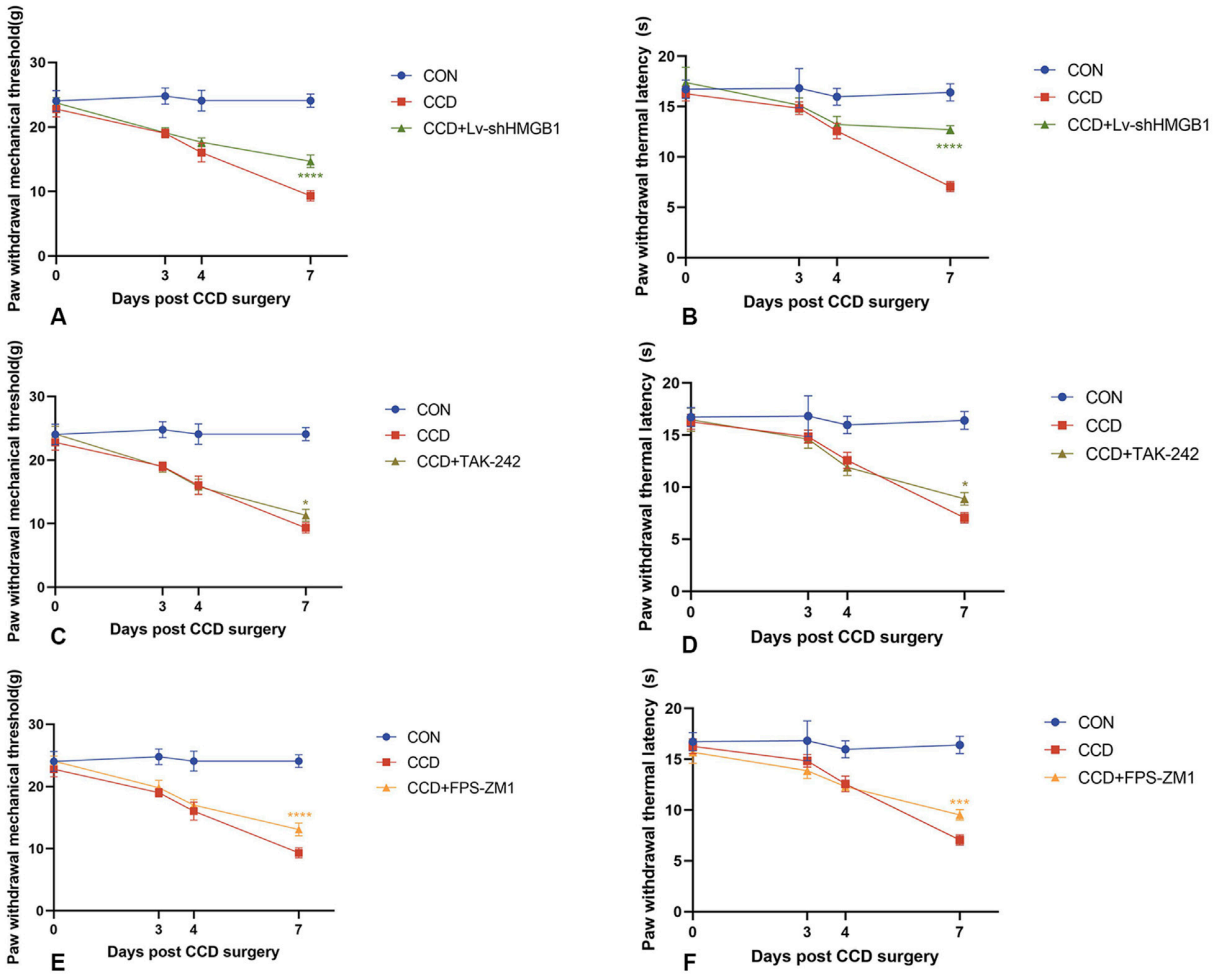
Mechanical and thermal pain threshold responses in CCD rats within each group **(A)** Mechanical pain thresholds in CCD rats after HMGB1 knockdown. **(B)** Thermal pain thresholds in CCD rats after HMGB1 knockdown. **(C)** Mechanical pain thresholds in CCD rats after TLR4 inhibition. **(D)** Thermal pain thresholds in CCD rats after TLR4 inhibition. **(E)** Mechanical pain thresholds in CCD rats after RAGE inhibition. **(F)** Thermal pain thresholds in CCD rats after RAGE inhibition. N = 5 per group ****p < 0.0001 ***p < 0.001 **p < 0.01*p < 0.05 vs. CCD.

Three consecutive days of intrathecal lentivirus were administered to CCD rats to ensure the efficacy of HMGB1 knockdown. Mechanical and thermal pain thresholds were then determined in these CCD rats to evaluate the impact of HMGB1 knockdown on nociceptive hypersensitivity. When tested on the seventh day following CCD surgery, rats in the HMGB1 lentiviral knockdown group showed statistically significant increase in mechanical and thermal pain thresholds when compared with the non-treated CCD rat group (*p* < 0.0001). These findings indicate that a reduction in HMGB1 expression exerts a considerable impact in alleviating NP in CCD rats (Figures 3A, B).

### 3.5 Presence of a link between HMGB1 and AQP 1

To determine whether an association exists between HMGB1 and AQP1, we constructed an E5062 HA-Aqp1 overexpression plasmid transfected with an E5078 Hmgb1-3flag overexpression plasmid and transfected the constructed plasmid into H293T cells. Expressions of HMGB1 and AQP1 were then determined in H293T cells using Flag- and HA-tagged antibodies.

**FIGURE 4:**
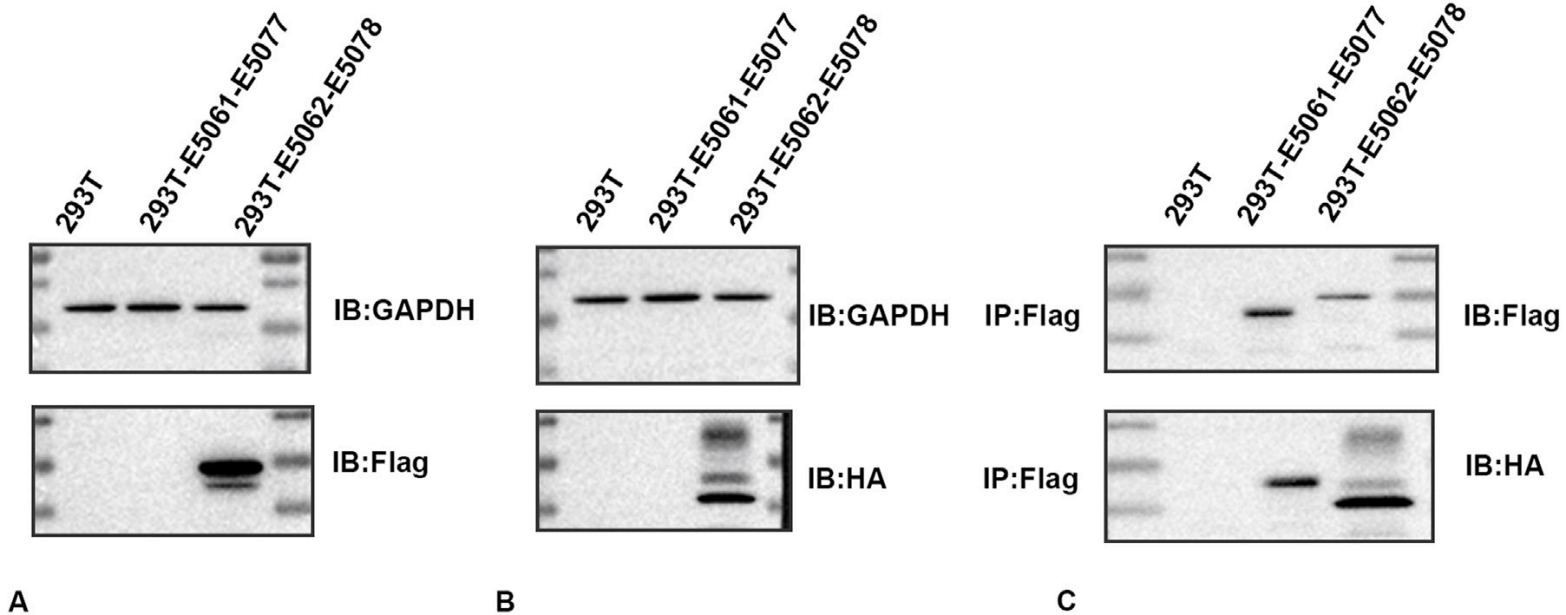
Immunoprecipitation determinations of HMGB1 and AQP1 **(A)** Western blots of HMGB1 in plasmids. **(B)** Western blots of AQP1 in plasmids. **(C)** CO-IP assay results. 293T: 293T-null cells; 293T-E5061-E5077: 293T- E5061 HA empty control plasmid transfection- E5077 negative control CON238 plasmid; 293T-E5062-E5078: 293T- E5062 HA-Aqp1 overexpression plasmid transfection- E5078 Hmgb1-3flag overexpression plasmid. Flag: HMGB1; HA:AQP1.

The HMGB1 protein was detected between the 15–35 KDa marker as observed using the Flag-tagged antibody (Figure 4A), while the AQP1 protein was detected between the 25–40 KDa marker using the HA-tagged antibody (Figure 4B). No expression of the target protein was observed in the negative control group, and the plasmid construction was successful. As shown in the CO-IP assay, the Flag-tagged antibody enriched AQP1 protein in rat astrocytes. The results of this CO-IP assay indicated that HMGB1 showed a direct or indirect interaction with AQP1 (Figure 4C).

### 3.6 HMGB1 knockdown reduces AQP1 expression and alleviates NP in CCD rats

**FIGURE 5:**
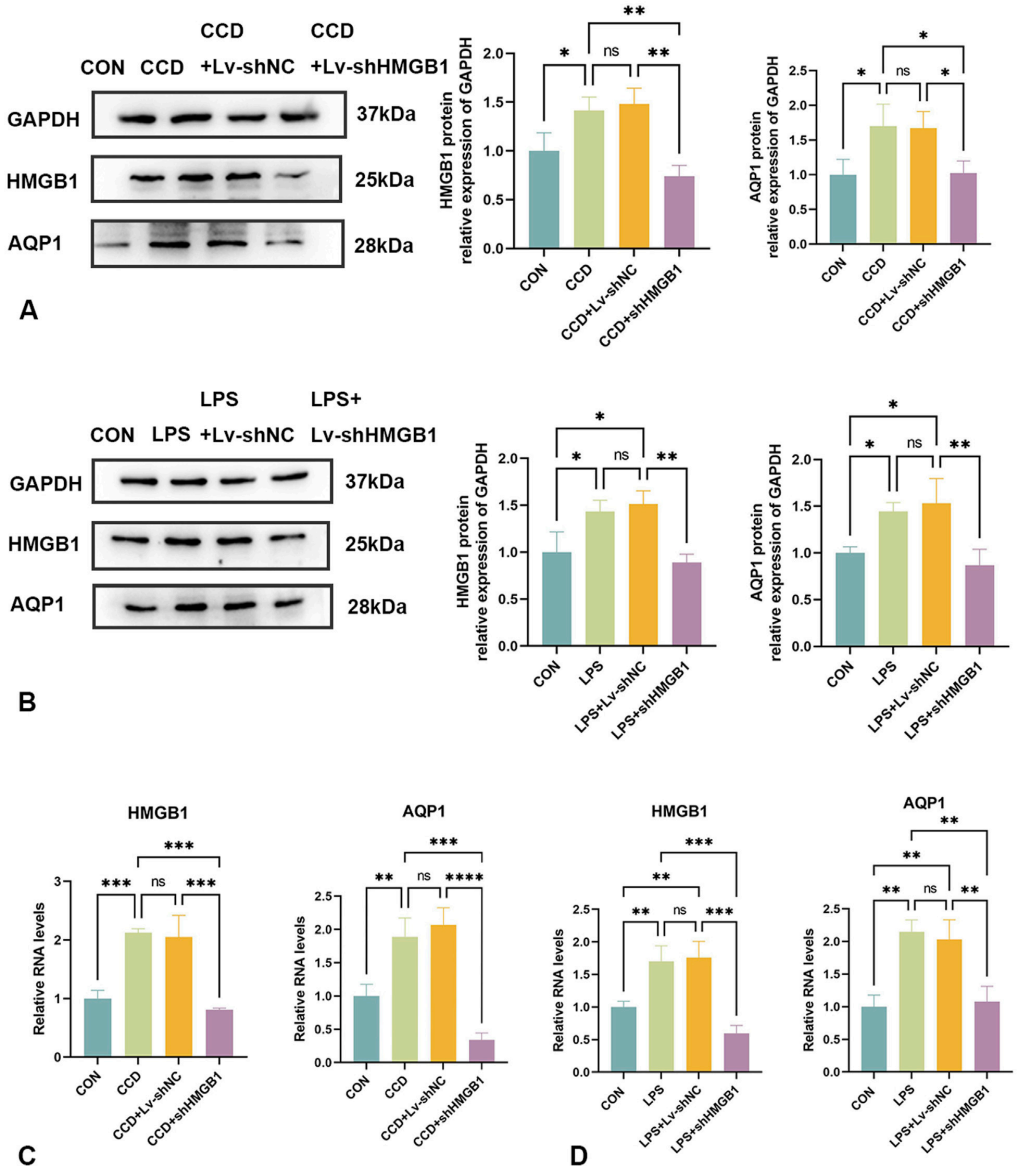
Changes in AQP1 expression after HMGB1 knockdown **(A)** Protein expression levels of HMGB1 and AQP1 in the spinal cords of rats in each group. **(B)** Protein expression levels of HMGB1 and AQP1 in the LPS inflammatory cell model in each group. **(C)** mRNA levels of HMGB1 and AQP1 in the spinal cords of rats in each group. **(D)** mRNA levels of HMGB1 and AQP1 in the LPS inflammatory cell model in each group. N = 3 per group ****p < 0.0001 ***p < 0.001 **p < 0.01*p < 0.05.

Protein and mRNA expressions of HMGB1 and AQP1 were significantly elevated in the spinal cords of CCD rats, while differences between the negative control group and the non-treated CCD group were not statistically significant (*p* > 0.05). With the silencing of HMGB1, there was a notable reduction in the protein expression of HMGB1 within the spinal cord of CCD rats as revealed using WB. Interestingly, there was also a notable reduction in spinal cord AQP1 protein expression in these HMGB1-silenced rats (Figure 5A). As based on the results from our PCR assay, the silencing of HMGB1 expression led to a notable reduction in the expressions of both HMGB1 and AQP1 mRNA in the spinal cord of CCD rats. (Figure 5C).

Similar results were observed within our *in vitro* cellular model. In response to LPS stimulation of rat astrocytes, expression levels of both HMGB1 and AQP1 protein (Figure 5B) and mRNA (Figure 5D) were elevated. After silencing HMGB1 expression in these cells, AQP1 expression was also significantly decreased (Figures 5B, D). Accordingly, as demonstrated in both *in vivo* and *in vitro* models, the inhibition of HMGB1 results in a corresponding reduction of AQP1 expression.

### 3.7 Inhibition of the HMGB1 receptor, TLR4, fails to alter AQP 1

To determine whether HMGB1 affects AQP1 expression through the TLR4 receptor, we tested the effects of inhibition of this receptor on AQP1 expression.

**FIGURE 6:**
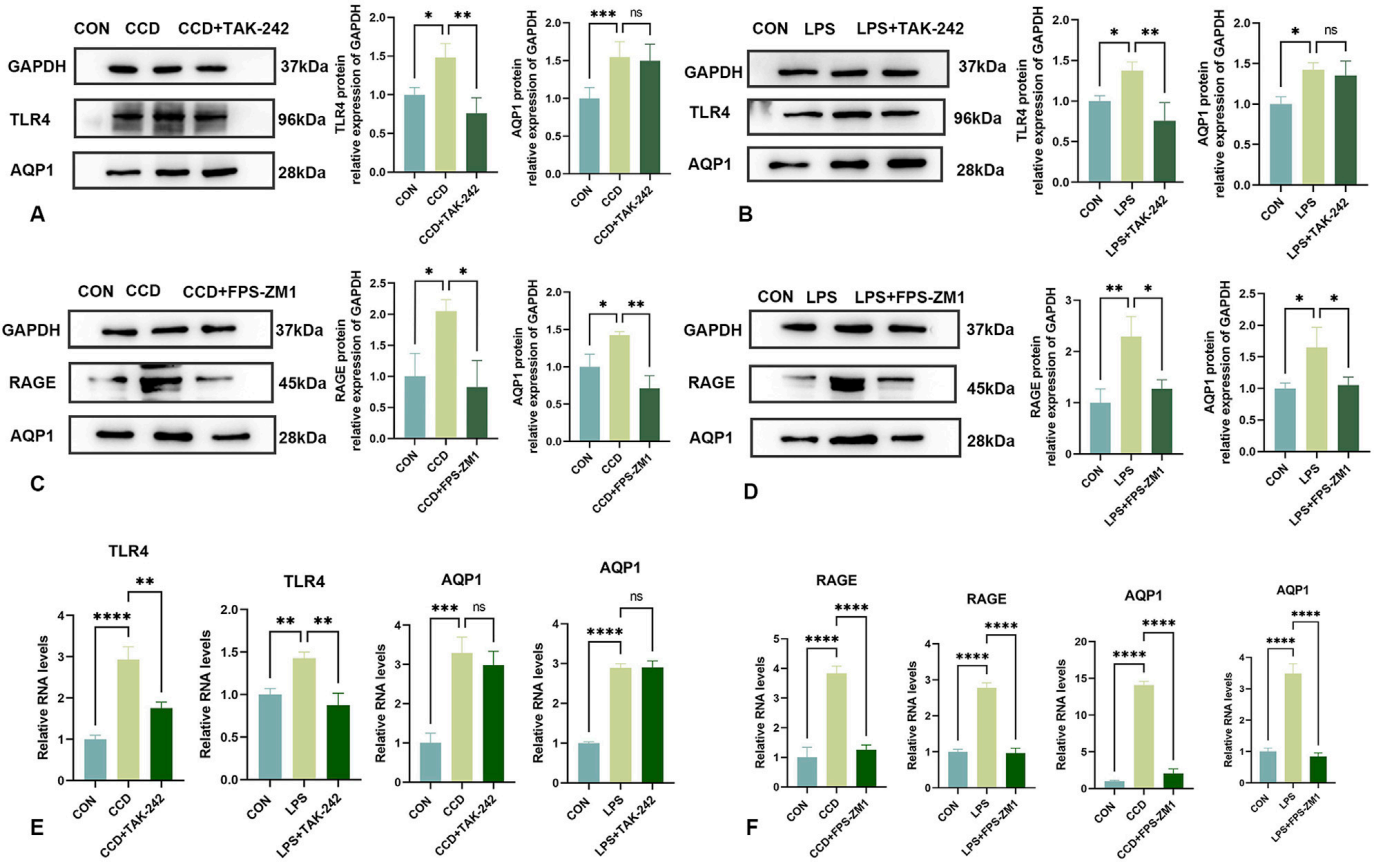
Changes in AQP1 expression after TAK-242 and FPS-ZM1 treatments **(A)** Protein expression levels of TLR4 and AQP1 in the spinal cords of rats in each group. **(B)** Protein expression levels of TLR4 and AQP1 in the LPS inflammatory cell model in each group. **(C)** Protein expression levels of RAGE and AQP1 in the spinal cords of rats in each group. **(D)** Protein expression levels of RAGE and AQP1 in the LPS inflammatory cell model in each group. **(E)** mRNA levels of TLR4 and AQP1 in each group. **(F)** mRNA levels of RAGE and AQP1 in each group. N = 3 per group ****p < 0.0001 ***p < 0.001 **p < 0.01*p < 0.05.

After treatment with the TLR4 receptor inhibitor, TAK-242, there were increases in mechanical and thermal pain thresholds in CCD rats as compared to that of the non-treated CCD group (Figures 3C,D). The findings that a notable reduction in TLR4 receptor protein expression levels was observed within the spinal cord of CCD rats suggests that this inhibitor effectively diminished the expression of the TLR4 receptor. However, no significant alterations in the protein expression of AQP1 were present in these TAK-242-treated CCD rats (Figure 6A). While the expression of TLR4 mRNA in the spinal cord of TAK-242-treated CCD rats was significantly decreased compared to non-treated CCD rats, mRNA expressions of AQP1 failed to differ statistically between these two groups (Figure 6E).

As an approach to corroborate these *in vivo* findings, an analogous experiment was performed within our *in vitro* LPS-stimulated inflammation model. After application of TAK-242, TLR4 protein expression was significantly reduced in these cells, indicating that this treatment was effective in decreasing the TLR4 receptor. Notably, similar to that observed with our *in vivo* model, there were no statistically significant changes in levels of AQP1 protein (Figure 6B) or mRNA (Figure 6E) in response to this TAK-242 treatment. These findings indicate that the effects of HMGB1 on AQP1 expression do not appear to occur via the TLR4 receptor.

### 3.8 Inhibition of the HMGB1 receptor, RAGE, significantly reduces AQP1

Unlike that of treatment with TAK-242, intraperitoneal injection of the RAGE receptor inhibitor, FPS-ZM1, in CCD rats significantly reduced the protein expression levels of RAGE and AQP1 (Figure 6C). Significant increase in mechanical and thermal pain thresholds were obtained in these FPS-ZM1-treated CCD rats (Figures 3E,F). Results of our PCR assay also showed that the mRNA expression of both RAGE and AQP1 was significantly reduced in the spinal cord of CCD rats treated with FPS-ZM1 (Figure 6F).

Results, as obtained with our *in vitro* model, were in accord with that of these findings from our *in vivo* model. Specifically, after treatment with FPS-ZM1 in our *in vitro* LPS-stimulated inflammation model, protein (Figure 6D), as well as mRNA (Figure 6F) expression levels of both RAGE and AQP1, were significantly reduced. When collating the findings of these last two sets of experiments it seems that it is the RAGE, and not the TLR4, receptor that is responsible for mediating the regulation of AQP1 by HMGB1.

### 3.9 HMGB1 alters NF-κB expression

**FIGURE 7:**
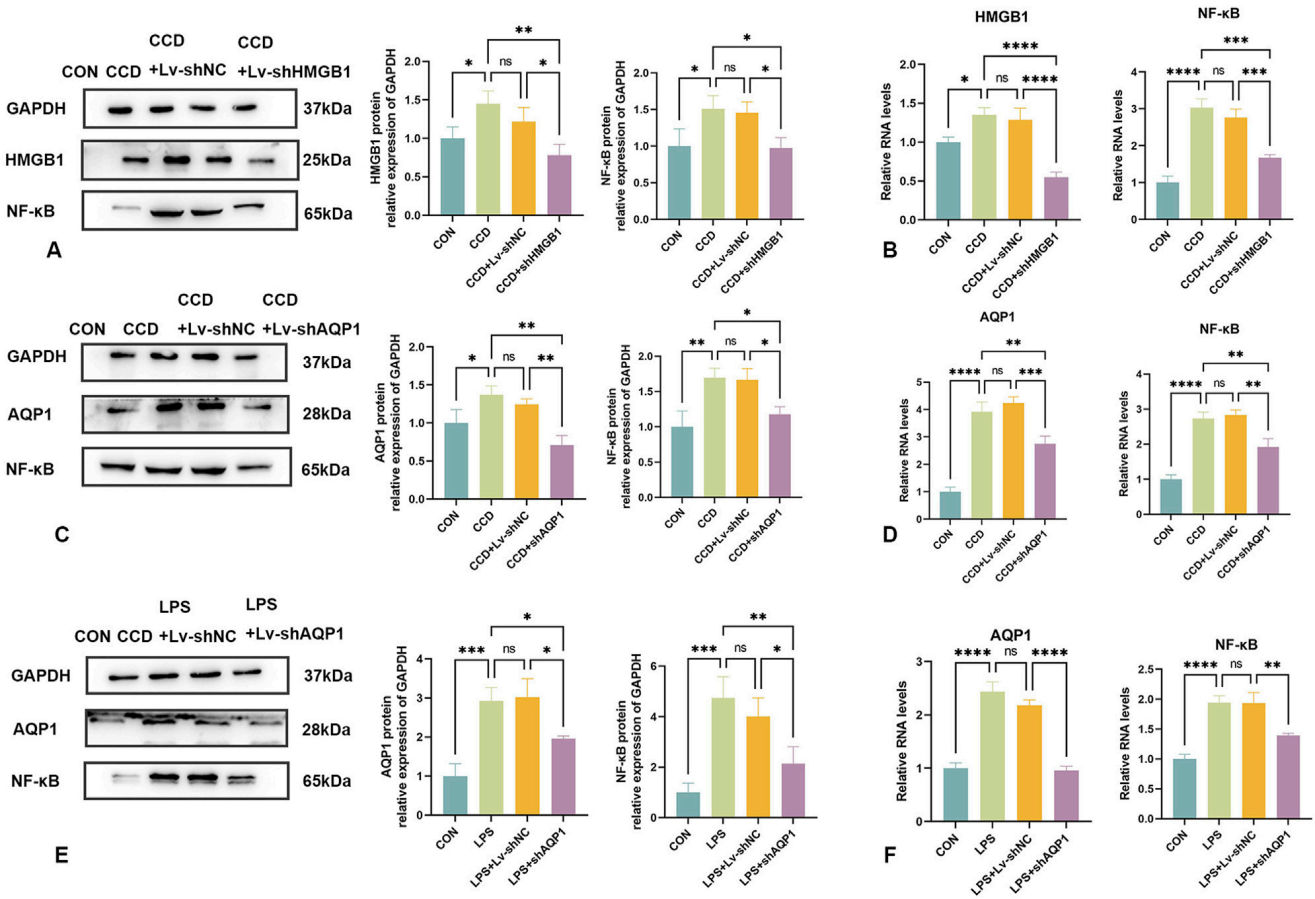
Changes in NF-κB expression following knockdown of HMGB1 or AQP1 **(A)** Protein expression levels of HMGB1 and NF-κB in the spinal cords of rats in each group. **(B)** mRNA levels of HMGB1 and NF-κB in the spinal cords of rats in each group. **(C)** Protein expression levels of AQP1 and NF-κB in the spinal cords of rats in each group. **(D)** mRNA levels of AQP1 and NF-κB in the spinal cords of rats in each group. **(E)** Protein expression levels of NF-κB and AQP1 in the LPS inflammatory cell model in each group. **(F)** mRNA levels of NF-κB and AQP1 in the LPS inflammatory cell model in each group. N = 3 per group ****p < 0.0001 ***p < 0.001 **p < 0.01*p < 0.05.

In this series of experiments, the role of NF-κB in NP was assessed. We found that spinal cord NF-κB protein expression was significantly elevated in CCD rats, but significantly decreased in these rats after HMGB1 knockdown (Figure 7A). While spinal cord mRNA expression of NF-κB was markedly elevated in CCD rats, a silencing of HMGB1 produced a notable decline in NF-κB mRNA expression (Figure 7B). Therefore, while NF-κB expression is markedly elevated in the spinal cord of CCD rats, a downregulation of HMGB1 produced a corresponding downregulation of NF-κB.

### 3.10 AQP1 knockdown reduces NF-κB expression

To evaluate the relationship between AQP1 and NF-κB in the spinal cord of CCD rats, expression levels of NF-κB were determined in these rats after knocking down AQP1.

The efficacy of AQP1 knockdown was verified by the notable reduction in AQP1 protein expression in the spinal cord of CCD rats. In these rats, there was a corresponding reduction in spinal cord NF-κB protein expression (Figure 7C). In addition, spinal cord mRNA expressions of both AQP1 and NF-κB in CCD rats were significantly decreased after knocking down AQP1, as demonstrated with PCR (Figure 7D). Accordingly, changes in the expression of AQP1 within CCD rats affects the expression of NF-κB.

Similarly, NF-κB protein and mRNA expression levels were significantly decreased with a knockdown of AQP1 in cells exposed to LPS-stimulated inflammation (Figures 7E, F). Collectively, these results show that in NP, AQP1 can function as an upstream regulator to modulate NF-κB expression.

## 4 Discussion

In this report, we find that HMGB1 modulates the expression of AQP1, which in turn affects NF-κB expression and ultimately NP. In animal experiments, silencing of HMGB1 was able to alleviate NP in a CCD rat model. Results from our CO-IP experiments revealed that a link exists between HMGB1 and AQP1 and that silencing of HMGB1 was followed by a decrease in the expressions of AQP1 and NF-κB. We also found that this regulatory effect of HMGB1 appears to be specifically mediated through its RAGE receptor. Silencing of AQP1 resulted in a significant decrease in NF-κB, indicating that a regulatory relationship is present between AQP1 and NF-κB. These results, as demonstrated in an *in vivo* CCD rat model of NP, were all corroborated in the cellular experiments as conducted in a LPS stimulated astrocytic inflammation model. Accordingly, the findings as obtained from these two models reveal some of the novel mechanisms regarding the role of HMGB1 in NP and suggest the potential for the development of a lentivirus-mediated pharmacological targeting of HMGB1 for use in the treatment of NP.

A particularly significant finding of this study was the identification of some of the novel mechanisms and role of HMGB1 in NP. NP involves an extremely complex and pathogenetically diverse disease process that is difficult to treat and rehabilitate with a single protocol (Ellis and Bennett, 2013; Rugnath et al., 2024). Since its initial characterization in 1972, HMGB1 and its functions in the nucleus, cytoplasm, cell membrane, and extracellular space have been subjected to continuous investigation (Tang et al., 2023). While nuclear HMGB1 maintains its chromosomal structure and function, during cellular stress or injury, HMGB1 is actively secreted and passively released into the extracellular compartment where it binds to its receptors and participates in inflammatory and immune responses (Chen et al., 2022). The results of our behavioral tests showed that nociceptive hypersensitivity was attenuated in CCD rats after silencing HMGB1 (Figures 3A,B). And, in a spinal nerve ligation (SNL) model, silencing of HMGB1 was also able to alleviate NP in SNL rats (Presto et al., 2023). Two important receptors for HMGB1 include RAGE and TLR4 (Yang et al., 2020). The HMGB1/TLR4/NF-κB pathway is involved in inhibiting microglial activation and neuroinflammation in mice (Beutler, 2004; Xu et al., 2020; Wang and Liu, 2021; Zhang et al., 2021) and, in mouse models of spinal cord injury or bone pain, inhibition of the HMGB1/RAGE/NF-κB pathway has been shown to alleviate neuroinflammation and reduce pain levels (Fan et al., 2020; Kim et al., 2021; Okui et al., 2021). These findings are consistent with the results of our current study. In specific, we showed that HMGB1, RAGE, TLR4, and NF-κB expressions were all upregulated in the spinal cord of CCD rats, while knockdown of HMGB1 or use of its receptor inhibitors significantly reversed these upregulations and alleviated NP in rats (Figures 5, 6). Similar effects were observed within our *in vitro* cellular experiments.

Based on current research, these effects appear to be achieved via a passive release of HMGB1 into the extracellular space. Results from our HMGB1 knockdown experiment suggest that nuclear HMGB1 is similarly affected. Nuclear HMGB1 can bind to export protein 1 (XPO1), become acetylated in response to oxidative stress and then translocate to the cytoplasm (Kwak et al., 2020). Therefore, we hypothesized that nuclear HMGB1 may be indirectly involved in the development of NP through other related modifications such as acetylation. While this conclusion needs to be substantiated with future studies, it seems clear that HMGB1 and its receptor occupy a pivotal role in NP. In the next series of investigations, we directed our efforts at identifying some of the mechanisms through which HMGB1 exerts these effects. Results from our CO-IP assay as well as from the silencing of HMGB1 revealed an association between AQP1 and HMGB1. Specifically, our results provide novel evidence indicating that AQP1 represents a downstream regulator of HMGB1. Therefore, we investigated this relationship between AQP1 and HMGB1 as related to NP.

While only a very limited amount of information had been presented regarding a link between HMGB1 and AQP1, a review of the literature over the past 5 years has provided some hints that HMGB1 was associated with AQPs. For example, Liu et al. (2024) reported that in a rat osteoarthritis (OA) model, Jianpi Tongluo Formula (JTF) was able to reduce osteoarthritic edema as well as HMGB1 and AQP1 expressions in these OA rats. Such effects reveal cartilage-protective and antidematogenic effects and findings from their network pharmacological analysis showed that the NCOA4-HMGB1-GSK3B-AQPs axis may serve as a new target for osteoarthritis treatment. As we also observed a link between HMGB1 and AQP1, as based on our CO-IP assay results (Figure 4), we then directed our efforts toward investigating this link as related to NP and the corresponding mechanisms and pathways involved. When applying the RAGE receptor to inhibit RAGE expression, we found that AQP1 was significantly downregulated, while no changes in AQP1 were obtained after the application of the TLR4 receptor inhibitor (Figure 6). These findings reveal that the RAGE receptor specifically mediates the regulation of AQP1 by HMGB1 in NP.

Aquaporins (AQPs), are a class of water-transporting proteins that play a role in fluid transport in cell types such as epithelial and endothelial cells, as well as in other cells (Verkman, 2002; Garcia et al., 2023). AQPs are involved in water movement, cell migration, and neural excitation in the central and peripheral nervous systems (Papadopoulos and Verkman, 2013). The first member of the water channel protein family to be discovered was AQP1, which is mainly distributed in the choroid plexus epithelium of the nervous system, the dorsal horn of the spinal cord, the small diameter sensory neurons of the dorsal root ganglion, as well as being expressed in the sciatic nerve of rats and mice (Preston and Agre, 1991; Shields et al., 2007; Segura-Anaya et al., 2022; Xiao et al., 2023). It has been reported that AQP1 knockout mice demonstrate a reduced responsiveness to chemical stimuli such as heat and capsaicin (Oshio et al., 2006). Such findings can be related to our current results which show that AQP1 expression was significantly increased in the spinal cord of CCD rats, effects which were accompanied by an enhanced degree of nociceptive sensitization and were associated with the development of NP (Figure 5). Intriguingly, our current experimental results have revealed that both AQP1 and HMGB1 exert influences on the expression levels of NF-κB. (Figures 7C, D). In support of our results is the study of Kalita et al. indicating a potential role for AQPs in LPS-stimulated inflammatory responses in a murine macrophage cell line (RAW264.7) and a human monocyte cell line (THP-1). Specifically, these investigators found a significant 2.14-fold increase in AQP1 protein along with an increase in mRNA expression (*p* < 0.05) at 24 h after LPS treatment in RAW 264.7 cells (Kalita and Das, 2024). Our current findings are in accord with the results of Kalita et al., as we observed that AQP1 knockdown significantly decreased spinal cord AQP1 and NF-κB expression along with alleviating NP in CCD rats. Such findings suggest that AQP1 seems to play a regulatory role in the inflammatory responses to NP as observed in this CCD model (Figures 7E, F). As NF-κB is a central mediator of inflammation, its activation may further regulate inflammatory factors such as interleukin 1β (IL-1β) and interleukin 6 (IL-6), which are critically involved in inflammatory responses (Roberti et al., 2022). Therefore, we hypothesized that both AQP1 and HMGB1 are closely associated with neuroinflammation, while changes in specific inflammatory markers will need to be clarified in future studies.

While the findings of this study offer some important new insights into HMGB1, its mechanisms, and pathways, as related to NP, there exist limitations of this study that will require follow-up investigations. HMGB1 possesses both structural and conformational specificity and is divided into three structural domains: two positively charged DNA-binding structural domains, the A-box (9-79aa) and the B-box (95-163aa), and a negatively charged C-terminus (acidic tail 186-215aa) (Bianchi et al., 1992; Chen et al., 2022). The extracellular B-box mainly exerts pro-inflammatory effects, whereas the A-box has anti-inflammatory effects (Ren et al., 2023). Although both RAGE and TLR4 belong to different binding sites associated with HMGB1, our current results suggest that only RAGE may mediate the regulatory effects of HMGB1 on AQP1. However, we cannot exclude the possibility that other factors at the binding site of HMGB1 may mediate the regulation of AQP1 by HMGB1, which will require further in-depth studies.

Our results can be summarized into four major areas. First, silencing HMGB1 expression significantly attenuates nociceptive hypersensitivity in rats, suggesting that HMGB1 may represent a particularly critical target for pharmacological exploration in the treatment of NP. Second, HMGB1 and AQP1 are somehow linked, as related to NP, with the silencing of HMGB1 leading to a decrease in the expression of AQP1 and the inflammatory pathway-related factor, NF-κB. Accordingly, AQP1 can be regarded as a downstream regulator of HMGB1 involved in the regulation of neuroinflammation in NP. Third, RAGE serves as a specific receptor for HMGB1 as being involved with mediating the regulation of AQP1. Fourth, based on the above conclusions, we can clarify some of the upstream-downstream relationships between AQP1 and NF-κB, with the silencing of AQP1 being a significant factor in affecting the expression of NF-κB. Collectively, these findings reveal an important role for an RNA interference gene therapy targeting HMGB1 in the alleviation of NP. Notably, the HMGB1-RAGE-AQP1-NF-κB pathway may provide new pharmacological targets for NP-related RNA interference gene therapy. Overall, the regulatory relationship between HMGB1 and AQP1 may suggest a critical connection between late inflammatory factors and the water channel protein family, a relationship warranting future investigations.

## 5 Conclusion

In an *in vivo* model of NP, lentivirus-mediated RNA interference gene therapy targeting HMGB1 was effective in alleviating this NP. Silencing of HMGB1 downregulated the expression of AQP1, which in turn led to a decrease in the expression of NF-κB, ultimately alleviating nociceptive hypersensitivity in this *in vivo* CCD rat model of NP. This modulation of AQP1 by HMGB1 is likely mediated through its RAGE receptor. Similar results were obtained with use of an *in vitro* LPS-simulated inflammation astrocytic cell model of NP.

## Data Availability

The original contributions presented in the study are included in the article/supplementary material, further inquiries can be directed to the corresponding author.

## Abbreviations

NP, Neuropathic pain; CCD, chronic compression of the dorsal root ganglia; PWMT, paw withdrawal mechanical threshold; TPWL, thermal paw withdrawal latency; HMGB1, High mobility group box 1; AQP1, aquaporin 1; shRNA, short hairpin RNA; CO-IP, Co-immunoprecipitation; CNS, central nervous system; RAGE, the Receptor of Advanced Glycation Endproducts; TLR4, Toll-like receptor 4; CCI, chronic constrictive injury; DRG, dorsal root ganglia; NF-κB, Nuclear factor kappa-B.
